# An AP Endonuclease 1–DNA Polymerase β Complex: Theoretical Prediction of Interacting Surfaces

**DOI:** 10.1371/journal.pcbi.1000066

**Published:** 2008-04-25

**Authors:** Alexej Abyzov, Alper Uzun, Phyllis R. Strauss, Valentin A. Ilyin

**Affiliations:** Department of Biology, Northeastern University, Boston, Massachusetts, United States of America; Stanford University, United States of America

## Abstract

Abasic (AP) sites in DNA arise through both endogenous and exogenous mechanisms. Since AP sites can prevent replication and transcription, the cell contains systems for their identification and repair. AP endonuclease (APEX1) cleaves the phosphodiester backbone 5′ to the AP site. The cleavage, a key step in the base excision repair pathway, is followed by nucleotide insertion and removal of the downstream deoxyribose moiety, performed most often by DNA polymerase beta (pol-β). While yeast two-hybrid studies and electrophoretic mobility shift assays provide evidence for interaction of APEX1 and pol-β, the specifics remain obscure. We describe a theoretical study designed to predict detailed interacting surfaces between APEX1 and pol-β based on published co-crystal structures of each enzyme bound to DNA. Several potentially interacting complexes were identified by sliding the protein molecules along DNA: two with pol-β located downstream of APEX1 (3′ to the damaged site) and three with pol-β located upstream of APEX1 (5′ to the damaged site). Molecular dynamics (MD) simulations, ensuring geometrical complementarity of interfaces, enabled us to predict interacting residues and calculate binding energies, which in two cases were sufficient (∼−10.0 kcal/mol) to form a stable complex and in one case a weakly interacting complex. Analysis of interface behavior during MD simulation and visual inspection of interfaces allowed us to conclude that complexes with pol-β at the 3′-side of APEX1 are those most likely to occur *in vivo*. Additional multiple sequence analyses of APEX1 and pol-β in related organisms identified a set of correlated mutations of specific residues at the predicted interfaces. Based on these results, we propose that pol-β in the open or closed conformation interacts and makes a stable interface with APEX1 bound to a cleaved abasic site on the 3′ side. The method described here can be used for analysis in any DNA-metabolizing pathway where weak interactions are the principal mode of cross-talk among participants and co-crystal structures of the individual components are available.

## Author Summary

Oxidative damage to DNA happens in every cell as a consequence of the life process. Such damage can inhibit DNA replication and RNA transcription; if not repaired, it can lead to cancer. Consequently, all cells contain an important mechanism for identification and repair of oxidative lesions. Two proteins figure prominently: AP endonuclease 1, which cleaves the damaged site, and DNA polymerase beta, which inserts a new nucleotide to replace the damaged one. While several biochemical studies indicate interaction between the two proteins, the details of the interaction remain unknown. Here, we develop and apply a new methodology to predict the most likely protein-protein interface between the two proteins. The methodology relies on the assumption, which is validated by experimental evidence, that both proteins must bind to DNA in order to interact. Analysis of the simulated protein behavior in water allowed us to suggest how protein interaction might be coupled to conformational changes in DNA polymerase beta. Further comparative analysis identified coordinated mutations of specific residues at the predicted interfaces. This method can be applied to predict interaction details for any protein pair as long as the proteins in the pair are associated with DNA during the interaction.

## Introduction

Loss of a nucleobase without cleavage of the DNA backbone results in formation of an abasic (AP) site. AP sites arise frequently in normal DNA from a variety of causes: spontaneous hydrolysis of nucleobases, DNA damaging agents or DNA glycosylases that remove specific abnormal bases, such as uracil, *N3*-methyladenine, or 8-oxoguanine. Since AP sites are pre-mutagenic lesions that can prevent normal DNA replication and transcription, the cell contains systems to identify and repair such sites, specifically the base excision repair (BER) pathway [1]. Apurinic/apyrimidinic endonuclease 1 (APEX1) cleaves the phosphodiester backbone 5′ to the AP site [2]–[4]. The cleavage, which is a key step in the BER pathway, is followed by nucleotide insertion and removal of the downstream deoxyribose moiety, performed most often by DNA polymerase beta (pol-β) [5]. The fact that nucleotide insertion requires cleavage of the AP site suggests interaction of the two enzymes.

Biological experiments to examine whether APEX1 and pol-β interact have been carried out using several different methodologies [6]–[11]. A complex of the two proteins was detected not only by yeast-two hybrid studies, but also by electrophoretic mobility shift assay (EMSA) and EMSA supershift followed by immunoblotting [6],[11]. In the latter studies the complex was detected only when DNA containing an uncleaved AP site was present. Furthermore, in kinetic studies the presence of APEX1.pol-β.DNA ternary complex stimulates pol-β gap filling activity [11]. The current model for a substrate containing a single nucleotide gap in double stranded DNA suggests that DNA binding specificity of APEX1 and pol-β determines the orchestrated coordination of the sequential steps, although a multiprotein–DNA complex facilitates coordination. The fact that the evidence for coordination of both enzymes requires the presence of substrate, i.e., AP-site containing DNA, suggests that the two proteins must be seated on the DNA in proximity to each other or that binding by APEX1 to the AP-site recruits the second protein.

We present detailed theoretical analysis of possible complexes between APEX1 and pol-β, at which EMSA analysis or the yeast two-hybrid system can only hint. The analysis predicts a communicative interaction between the two proteins under the assumption that the initial interaction occurs when both proteins are seated on the DNA helix. Crystal structures of the individual proteins bound to DNA are known [12],[13]. In this study the two proteins are positioned on a DNA helix to examine possible placements for interactions between them. Subsequent Molecular Dynamic (MD) simulation is applied to the complexes in order to ensure optimal atom packing on the protein-protein interface and identify interacting reisdues. Having identified critical amino acid residues at the interface, we propose a mechanism by which pol-β might displace APEX1 as the former enzyme seats itself at the cleaved abasic site.

## Results

**Figure 1:**
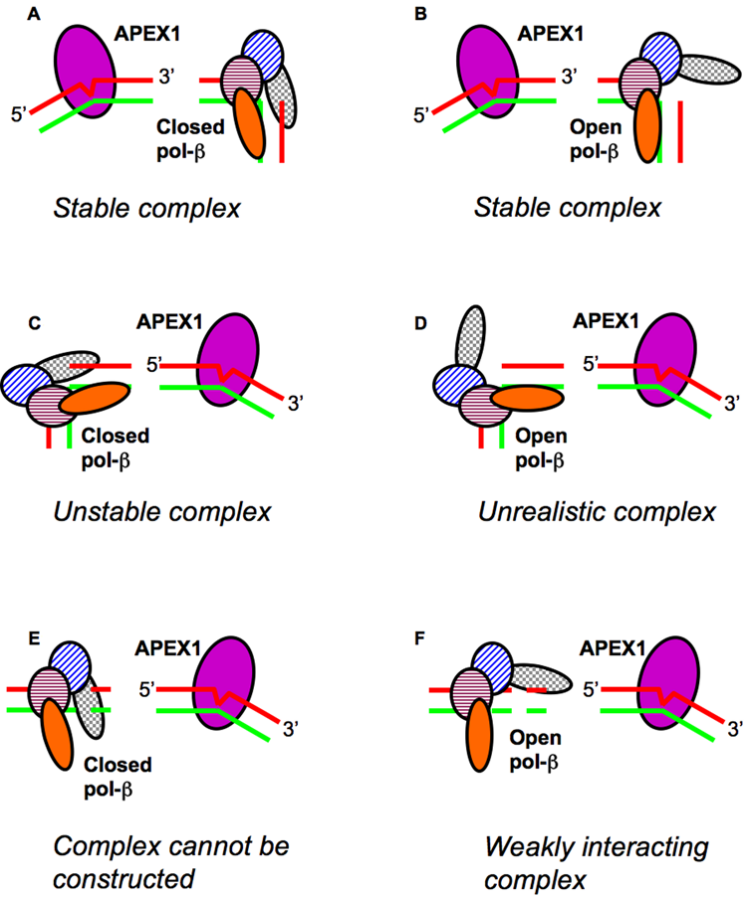
Schematic diagram of initial complex construction. Comments underneath each picture reflect the conclusion made about each complex after analysis.

A model of the initial complex was built by aligning the DNAs that were co-crystallized in complexes with APEX1 or with pol-β. The DNA strand in the co-crystal with APEX1 has a 30 degree bend at the AP site; therefore, in the initial complex we aligned the DNA in the pol-β co-crystal with 5′- or 3′-sides of the damaged DNA in the APEX1 co-crystal. Several potential interacting complexes from either side were considered (see schematic diagram in Figure 1).

We presumed that protein-protein interaction occurs when both proteins are associated with DNA, but that only one protein at a time performs its function at the damaged site. Since pol-β follows APEX1 in the BER pathway, it is likely to sense the cleaved DNA site through APEX1 bound to DNA. Therefore, the first priority was to correctly represent the interaction of APEX1 with DNA in the complex rather than that of pol-β. Consequently, we kept only DNA from the APEX1 co-crystal for subsequent molecular dynamics. The 90 degree bend found in the pol-β co-crystal will not be present in the initial complex.

Crystal structures for pol-β are available in three different conformations: one that allows for the incoming nucleotide to correctly hydrogen bond in preparation for insertion [14] (here called the open conformation); a precatalytic state with bound nucleotide [13] (here called the closed conformation); and the intermediate state (not used in this study). At first we constructed and analyzed the complexes with pol-β in the closed conformation and then we extended our study by constructing and analyzing similar complexes with pol-β in the open conformation.

### Complex at the 3′ Side of APEX1 with pol-β in Closed Conformation

**Figure 2:**
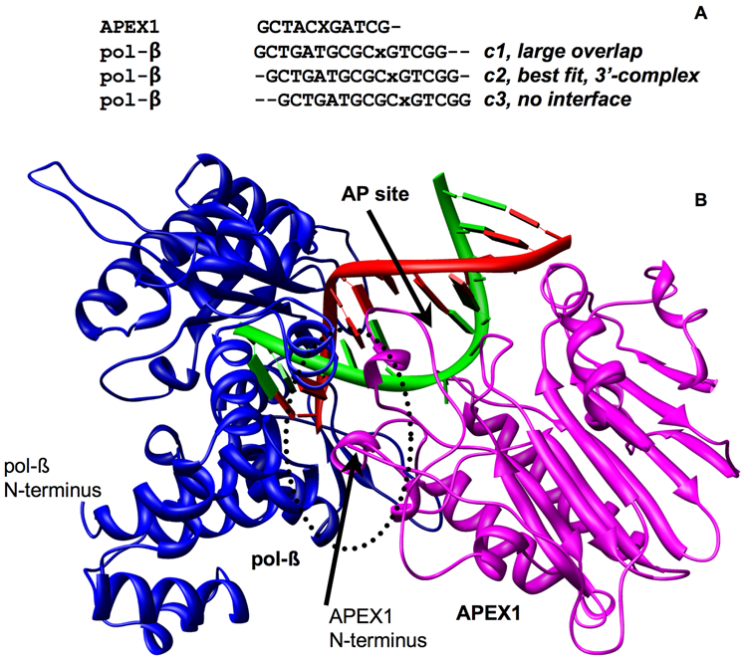
Initial 3′ complex of APEX1 and pol-β (closed conformation). (A) Alignment of DNAs co-crystallized with pol-β and APEX1. X stands for the abasic site and x stands for lesion. Notations c1, c2, and c3 mark the alignments used to produce three initial complexes. (B) View of the 3′ complex structure. APEX1 is on the right and pol-β is on the left. The area of protein-protein interaction is circled.

For this orientation (see schematic diagram in Figure 1A), initial complexes for pol-β in the closed conformation (PDB-file 2fmq) and APEX1 (PDB-file 1de8) were constructed by aligning the 3′-side of damaged DNA from the APEX1 co-crystal with the 5′-side of the DNA lesion in the pol-β co-crystal. Three complexes termed c1, c2, and c3 satisfied the described requirements (see corresponding alignment in Figure 2A). In the first complex (c1), steric overlaps between APEX1 and pol-β involved more than 10 residues comprising more than 100 atoms in each protein. Polypeptide chains of the two proteins interlaced with each other to produce an unrealistic complex. In the third complex (c3) the interface area was ∼200 Å^2^ but the ratio of gap volume to area (∼140) was unacceptably large compared to other values in Table 1, indicating very weak interaction, if any. The remaining complex (c2) represented an optimal prediction with only several atoms in steric overlap, which were resolved during MD (see below). The complex is shown in Figure 2B.

**Table 1:** Parameters of protein-protein interfaces for the 3′ complex (with pol-β in closed conformation).

Time (ns)	Interface Area (Å^2^)	Interface Area (% of Surface area)	Planarity RMSD (Å)	Length & Breadth (Å)	Polar Atoms (% of interface)	Non-polar Atoms (% of Interface)	Gap Volume (Å^3^)	Gap Volume/Interface Area	Hydrogen Bonds at Interface	Salt Bridges in Interface	Binding Energy (kcal/mol)	Area of segment #1 (Å^2^)	Area of segment #2 (Å^2^)	Area of segment #3 (Å^2^)
0.1	565	4.2	2.7	37 & 24	47	53	8655	7.5	6	0	−6.6	142	238	185
0.2	644	4.7	3.7	40 & 25	50	50	8731	6.7	3	0	−6.6	159	281	204
0.3	530	3.8	3.1	37 & 24	49	51	8480	7.9	2	0	−2.8	140	227	163
0.4	521	4.0	2.9	27 & 24	43	57	8894	8.3	5	0	−8.3	166	191	164
0.5	544	4.1	3.0	35 & 21	43	57	9681	8.5	6	0	−5.0	132	226	186
0.6	585	4.4	2.8	36 & 25	40	60	10060	8.4	5	0	−6.1	148	230	207
0.7	540	4.1	3.0	35 & 26	49	51	9465	8.6	5	0	−10.0	89	239	212
0.8	450	3.4	2.6	34 & 22	54	46	11862	12.8	3	0	−5.8	108	215	127
0.9	478	3.6	2.7	40 & 24	51	49	10089	10.3	4	0	−9.5	137	194	147
1.0	632	4.8	3.4	39 & 24	49	51	9879	7.7	5	0	−7.3	247	200	185

Values are calculated for interface in APEX1 after 0.1 ns intervals of MD simulation. To calculate the ratio of “gap volume/interface area,” the sum of interface areas in APEX1 and pol-β is used in denominator. The free energy of binding was calculated with FOLD-X server.

In order to resolve steric overlaps and ensure optimal atom packing at the protein-protein interface, an MD simulation was applied to the complex. The MD simulation continued for 1 ns (see Methods). The interface between APEX1 and pol-β was analyzed after each 0.1 ns of simulation (see Table 1).

During the entire simulation a stable complex was observed with interface area reaching 644 Å^2^ and binding energy as low as −10 kcal/mol. The interface area fluctuated by ∼30% while the shape of interface stayed essentially the same (see column ‘Length&Breadth’). Similarly, the interface atomic content did not change and consisted of slightly less than 50% of polar and slightly more than 50% non-polar atoms. On average the interface had 4 short-lived hydrogen bonds. Out of 21 different hydrogen bonds observed during the simulation at most 6 could be observed at any time. The most stable hydrogen bonds observed throughout the simulation contained the sidechain atoms of Asn^222^ and mainchain oxygen of Gln^31^ and mainchain nitrogen of His^34^. Estimation of binding energies with FOLD-X server revealed that the major contribution to the free energy of binding comes from hydrophobic desolvation and Van der Waals interaction with 1/3 contribution from hydrogen bonding. Thus, we concluded that the major interactions stabilizing the interface were hydrophobic.

**Figure 3:**
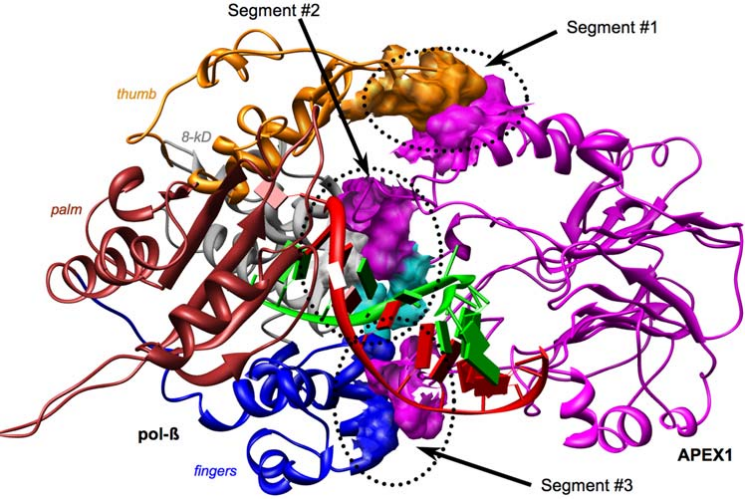
The interface of APEX1 and pol-β (closed conformation) in 3′ complex after 0.2 ns of MD simulation. The initial complex is shown in Figure 2. Subdomains of pol-β are colored by different colors and named. The protein-protein interface consists of three spatially distinct segments. Residues with correlated mutations (for segment #2: Arg^221^ of APEX1 and Gln^31^ of pol-β) are colored in cyan (see also Figure 6).

Analysis of the complex allowed identification of potential interacting residues of APEX1 and pol-β (see Table 2). The interface of APEX1 contained 16 residues with six, Arg^221^, Asn^222^, Lys^224^, Gln^235^, Ser^275^ and Lys^276^, representing the largest interface surface. The interface of pol-β contained 13 residues with seven, Gln^31^, Ile^33^, His^34^, Ser^109^, Lys^113^, Gly^305^ and Val^306^, contributing the largest interface area. Overall the interface consisted of three distinct spatial segments (Figure 3). In pol-β the interfaces of each segment were composed of residues from different subdomains: in segment #1 from the thumb subdomain, in segment #2 from the 8-kD subdomain and in segment #3 from the finger subdomain. Segments #1 and #3 were smaller then segment #2. The segments behave differently during the simulation (see Table 1). The areas of segments #2 and #3 were essentially stable while the area of segment #1 fluctuated (see Table 2). Not all of the amino acid residues in the segment #1 participated in interaction at all times.

**Table 2:** Interface residues of APEX1 and pol-β (closed conformation) in the 3′ complex.

Seg	Residues in APEX1	Interface Area (percentage)	Residues in pol-β	Interface Area (percentage)
1	Leu^179^	0–10	Arg^299^	0–5
	Leu^182^	4–7	Leu^301^	0–6
	Glu^183^	0–5	Val^303^	0–14
	Gln^186^	0–11	Thr^304^	0–11
	Pro^234^	0–7	**Gly^305^**	2–10
	**Gln^235^**	6–10	**Val^306^**	5–15
	Gln^238^	0–6	Ala^307^	0–5
2	**Arg^221^**	2–12	**Gln^31^**	7–14
	**Asn^222^**	9–16	**Ile^33^**	4–10
	Pro^223^	2–5	**His^34^**	16–26
	**Lys^224^**	5–17		
	Gly^225^	2–7		
3	Asn^272^	0–5	**Ser^109^**	6–11
	**Ser^275^**	6–12	Arg^112^	0–8
	**Lys^276^**	13–17	**Lys^113^**	10–13
	Asn**^277^**	1–7		

The residues assembling the majority of the interface are highlighted in bold. The columns “Seg” refers to the discrete interface segments where the listed residues are located (see Figure 3). The column “Interface Area” displays range of interface area represented by a residue during MD simulation (see Table 1).

### Complex at the 3′ Side with pol-β in Open Conformation

The 3′-complex with pol-β in open conformation (PDB-file 9ici) was constructed in the same fashion as the 3′-complex with pol-β in the closed conformation (see schematic diagram on Figure 1B). The MD simulation of the complex revealed critical differences in behavior of the interface (see Table 3). Namely the interface area increased steadily during the simulation and was on average 10% larger than that for the complex with pol-β in closed conformation, suggesting stronger binding, even though the estimated free energies of binding were similar. The fact that the interface was larger was remarkable since the protein-protein interface in the complex with pol-β in open conformation lacked segment #1.

**Table 3:** Parameters of protein-protein interfaces for the 3′ complex (with pol-β in open conformation).

Time (ns)	Interface Area (Å^2^)	Interface Area (% of Surface area)	Planarity RMSD (Å)	Length & Breadth (Å)	Polar Atoms (% of interface)	Non-polar Atoms (% of Interface)	Gap Volume (Å^3^)	Gap Volume/Interface Area	Hydrogen Bonds at Interface	Salt Bridges in Interface	Binding Energy (kcal/mol)
0.1	453	3.4	2.2	25 & 23	48	52	9600	10.3	2	0	−5.4
0.2	530	3.8	2.1	26 & 18	41	59	11612	10.7	1	0	−5.2
0.3	569	4.1	2.1	26 & 19	47	53	10579	9.2	2	0	−3.4
0.4	603	4.3	2.2	29 & 21	52	48	8305	6.7	3	0	−10.0
0.5	573	4.1	2.3	29 & 17	51	49	12766	10.7	6	0	−7.1
0.6	644	4.5	2.4	31 & 23	48	52	11227	8.4	5	0	−5.2
0.7	639	4.3	2.8	29 & 19	44	56	13134	10.0	5	0	−9.6
0.8	645	4.3	2.5	34 & 19	49	51	11145	8.5	3	0	−6.5
0.9	687	4.5	3.4	31 & 34	46	54	13799	10.0	4	0	−7.7
1.0	732	4.8	3.6	42 & 30	49	51	13752	9.4	3	0	−5.2

Values are calculated for interface in APEX1 after 0.1 ns intervals of MD simulation. To calculate the ratio of “gap volume/interface area” the sum of interface areas in APEX1 and pol-β is used in denominator. The free energy of binding was calculated with FOLD-X server.

For most of the simulation time the interface consisted of a single surface patch formed from segments #2 and #3 of the complex with closed conformation of pol-β plus several peripheral residues: Leu^44^, Glu^217^, Ile^218^, Asn^259^, Pro^261^, Tyr^262^, Tyr^264^ in APEX1 and Ala^32^, Arg^40^, Thr^93^, Val^115^, Glu^117^ in pol-β. In the last 0.1 ns of simulation another patch appeared between residues 177–183 in APEX1 and 231–233 in pol-β. Since its area was less than 50 Å^2^, we neglected it in subsequent analysis. The interface atomic content was similar to the one observed for complex with pol-β in closed conformation, i.e, it consisted of slightly less than 50% polar and slightly more than 50% non-polar atoms. At the same time there were fewer hydrogen bonds at the interface suggesting that hydrophobic interaction was even more important for interface stabilization than in the complex with pol-β in closed conformation.

### Complex at the 5′ Side of APEX1 with pol-β in Closed Conformation

**Figure 4:**
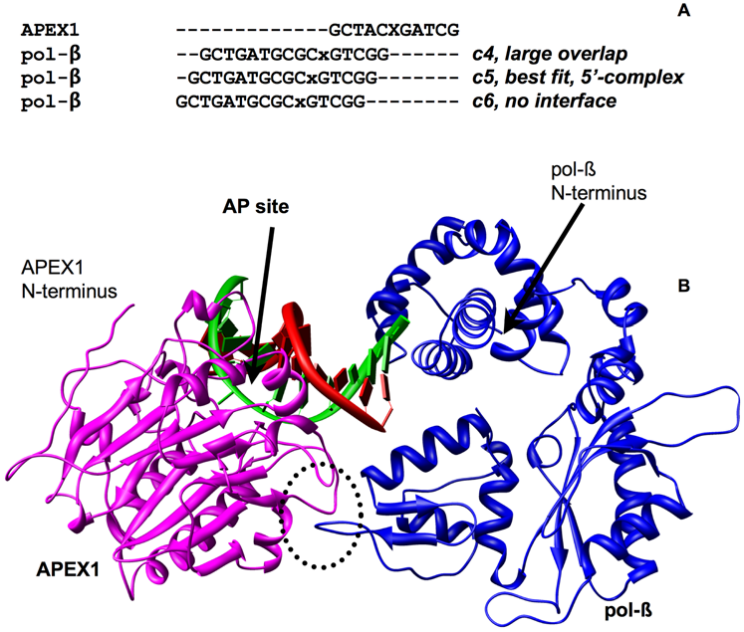
Initial 5′ complex of APEX1 and pol-β (closed conformation). (A) Alignment of DNAs co-crystallized with pol-β and APEX1. X stands for the abasic site and x stands for lesion. Notations c4, c5, and c6 mark the alignments used to produce three initial complexes. (B) View of the 5′ complex structure. APEX1 is on the left and pol-β is on the right. The area of protein-protein interaction is circled.

For this orientation (see schematic diagram on Figure 1C), three possible complexes using pol-β in the closed conformation and APEX1 were initially constructed by aligning the 5′-side of the damaged DNA from the APEX1 co-crystal with the 3′-side of the DNA with lesion in the pol-β co-crystal. Three complexes termed c4, c5, and c6 satisfied the described requirements (see corresponding alignment in Figure 4A). In the first complex (c4), steric overlaps of APEX1 and pol-β were large, involving more than 15 residues with more that 150 atoms in each protein. Moreover, polypeptide chains from the two proteins interlaced, producing an unrealistic complex. In the third complex (c6) the proteins hardly touched each other so that the corresponding interface was small with large water filled space between proteins. The remaining complex (c5) represented an optimal prediction with several atoms in steric overlaps, which were resolved during MD (see below). The complex is shown in Figure 4B.

MD was applied to the 5′-complex in order to resolve steric overlaps and ensure optimal atom packing at the protein-protein interface. The MD simulation continued for 1 ns (see Methods) and revealed an unstable, short-lived complex. Already after 0.1 ns of simulation the interface area was only 228 Å^2^ and after 0.4 ns the complex dissociated completely. Therefore, we concluded that the 5′-complex with pol-β in closed conformation was not likely to exist.

### Complex at the 5′ Side of APEX1 with pol-β in Open Conformation

The 5′-complex with pol-β in open conformation was constructed by replacing the structure of pol-β in the c4 complex (see Figure 4A) with pol-β in the open conformation (see Methods). Because replacement of pol-β in the c5 complex resulted in a complex lacking an interface, we used the c4 complex instead. In order to resolve steric overlaps and ensure optimal atom packing at the protein-protein interface, an MD simulation was applied to the c4 complex (see Table 4). During the entire simulation a complex with negative (favorable) free energies of binding and large interface area was observed. Despite the favorable energy of binding and the apparently large interface area, the interdigitated configuration of the interface suggests that the physical measurements were misleading. The interface of APEX1 (see Table 5) contained 17 residues with Ser^123^, Asp^124^, Lys^125^, and Gln^153^, representing the largest interface area. The majority of interface (60–70%) in pol-β was composed of the loop consisting of residues 299–306 from the thumb domain. Val^303^, Thr^304^ and Val^306^ of pol-β were buried into the APEX1 molecule hooked around APEX1 residues Tyr^144^ and Asp^152^. Furthermore, the interface dynamics (see Table 4) indicate that the interface is unstable. Thus, based on analysis of interface dynamics and visual inspection we concluded that the complex is not likely to exist.

**Table 4:** Parameters of protein-protein interfaces for the 5′ complex (with pol-β in open conformation).

Time (ns)	Interface Area (Å^2^)	Interface Area (% of Surface area)	Planarity RMSD (Å)	Length & Breadth (Å)	Polar Atoms (% of interface)	Non-polar Atoms (% of Interface)	Gap Volume (Å^3^)	Gap Volume/Interface Area	Hydrogen Bonds at Interface	Salt Bridges in Interface	Binding Energy (kcal/mol)
0.1	818	5.9	3.1	30 & 23	51	49	8963	5.4	5	0	−10.0
0.2	801	5.7	3.1	27 & 24	51	49	9757	5.9	6	1	−13.5
0.3	789	5.6	3.9	32 & 25	57	43	7156	4.4	8	1	−6.7
0.4	775	5.4	3.0	29 & 22	49	51	8847	5.5	6	1	−6.6
0.5	757	5.2	3.1	29 & 24	47	53	7978	5.2	8	0	−10.9
0.6	693	4.8	3.0	29 & 23	46	52	7762	5.4	8	0	−6.1
0.7	783	5.4	3.8	33 & 24	54	46	7337	4.5	8	0	−7.4
0.8	667	4.5	3.9	33 & 23	45	55	7622	5.4	5	0	−5.8
0.9	729	4.8	2.9	27 & 23	52	48	8707	5.9	6	0	−6.1
1.0	654	4.4	2.7	26 & 22	51	49	8235	6.1	6	0	−5.2

Values are calculated for interface in APEX1 after 0.1 ns intervals of MD simulation. To calculate the ratio of “gap volume/interface area” the sum of interface areas in APEX1 and pol-β is used in denominator. The free energy of binding was calculated with FOLD-X server.

**Table 5:** Interface residues of APEX1 and pol-β in the 5′ complex.

Residues in APEX1	Interface Area (percentage)	Residues in pol-β	Interface Area (percentage)
Glu^101^	0–6	**Arg^283^**	6–10
Asn^102^	0–5	Leu^287^	5–8
Ser^120^	0–5	Glu^288^	0–8
Ala^121^	0–6	Lys^289^	1–7
**Ser^123^**	5–11	Phe^291^	0–5
**Asp^124^**	12–14	Thr^292^	3–6
**Lys^125^**	6–10	Asn^294^	2–5
Glu^126^	2–6	Arg^299^	0–7
Tyr^144^	0–8	**Leu^301^**	5–12
Glu^149^	0–6	Gly^302^	3–5
Glu^150^	0–7	**Val^303^**	13–18
Asp^152^	3–6	**Thr^304^**	5–14
**Gln^153^**	12–17	Gly^305^	3–8
Glu^154^	0–5	**Val^306^**	13–18
Gly^155^	5–8		
Val^157^	4–7		
Arg^181^	0–5		

The residues assembling the majority of the interface are highlighted in bold. The column “Interface Area” displays range of interface area represented by a residue during MD simulation (see Table 4).

### Complex at the 5′ Side of APEX1 with pol-β in Open Conformation and Straight DNA

The 90 degree bend of DNA found in the co-crystals of pol-β reflects the state when pol-β is seated on the cleaved AP-site. Since details of its binding to DNA are unknown we considered the possibility that pol-β can bind to a straight DNA, displace APEX1 and occupy the site of the lesion. In order to explore this possibility we, extended the DNA in the pol-β co-crystal with a straight 12-mer template DNA taken from the PDB. We then constructed complexes in the same way as we did for the complexes described above, i.e., by aligning DNAs from pol-β and APEX1 co-crystals.

**Figure 5:**
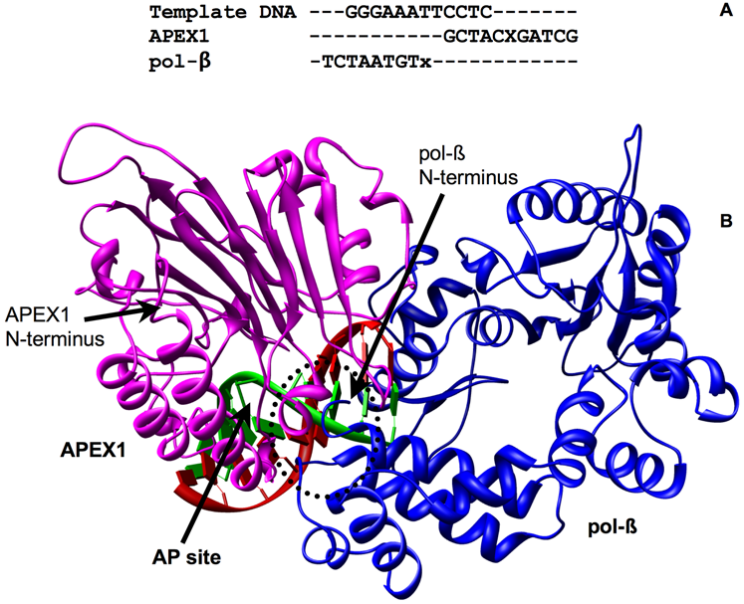
Initial 5′ complex of APEX1 and pol-β (open conformation) constructed on a straight DNA. (A) Alignment of 12-mer template DNA and DNAs co-crystallized with pol-β and APEX1. X stands for the abasic site and x stands for lesion. (B) View of the structure of the complex. APEX1 is on the right and pol-β is on the left. The area of protein-protein interaction is circled.

No new complexes at the 3′-side of APEX1 could be constructed, since the extended DNA intruded into pol-β. For the same reason no complex at the 5′-side of APEX1 with pol-β in closed conformation could be constructed (see Figure 1E). The only meaningful complex with straight DNA strand included pol-β in the open conformation located at the 5′-side of APEX1 (see Figure 1F). The DNA alignment and the complex are shown in Figure 5.

MD of the complex revealed weak interaction of pol-β and APEX1 (see Table 6). Over the time of simulation the interface area increased and binding energies improved (average of −4.3 kcal/mol) but was still less than the typical binding energy (<−10 kcal/mol) for crystallized protein-protein complexes [15]. The interface atom content consisted of more non-polar atoms than polar atoms. While 15 different hydrogen bonds were observed during the simulation, almost all of them were short lived, with at most 7 observed at a time. Only one hydrogen bond was preserved through out the simulation, between Lys^125^ of APEX1 and Asp^17^ pol-β. Estimation of binding energies with FOLD-X server revealed that the major contribution to the free energy of binding came from hydrophobic desolvation and Van der Waals interaction, while contribution of hydrogen bonding and electrostatic interactions (salt bridge) were two to three times smaller. Thus we concluded that the major interactions stabilizing the interface were hydrophobic.

**Table 6:** Parameters of protein-protein interfaces for the 5′ complex (with pol-β in open conformation and straight DNA).

Time (ns)	Interface Area (Å^2^)	Interface Area (% of Surface area)	Planarity RMSD (Å)	Length & Breadth (Å)	Polar Atoms (% of interface)	Non-polar Atoms (% of Interface)	Gap Volume (Å^3^)	Gap Volume/Interface Area	Hydrogen Bonds at Interface	Salt Bridges in Interface	Binding Energy (kcal/mol)
0.1	592	4.3	3.3	40 & 22	48	52	11652	10.0	5	0	−0.9
0.2	520	3.8	3.4	42 & 22	46	54	9425	8.9	7	0	−5.6
0.3	504	3.5	3.4	43 & 23	42	58	9418	9.2	3	1	−3.2
0.4	620	4.3	3.4	42 & 24	47	53	8806	7.0	5	1	−4.4
0.5	671	3.9	3.8	41 & 59	41	59	8276	7.0	6	1	−2.8
0.6	591	4.0	3.2	43 & 24	41	59	9664	8.1	4	1	−3.8
0.7	582	4.0	3.1	42 & 23	46	54	9092	7.6	5	1	−2.1
0.8	639	4.3	3.5	46 & 23	49	51	7459	5.7	5	1	−8.4
0.9	701	4.7	3.6	39 & 25	47	53	6829	4.8	6	1	−6.4
1.0	651	4.4	3.5	41 & 23	52	48	7150	5.5	5	1	−5.1

Values are calculated for interface in APEX1 after 0.1 ns intervals of MD simulation. To calculate the ratio of “gap volume/interface area” the sum of interface areas in APEX1 and pol-β is used in denominator. The free energy of binding was calculated with FOLD-X server.

Overall the interface consisted of one large and one small segment (Table 7). The residues from pol-β in the small segment were from the thumb domain, while residues in the large segment were from the fingers domain. The interface differed from that in the 3′-complex by being less planar and more elongated, despite similar surface areas (Tables 1 and 6). Otherwise this complex was substantially weaker in binding.

**Table 7:** Interface residues of APEX1 and pol-β (open conformation) in the 5′ complex with straight DNA.

Seg	Residues in APEX1	Interface Area (percentage)	Residues in pol-β	Interface Area (percentage)
Small	**Lys^77^**	**1–14**	Lys^331^	0–5
			Asp^332^	5–8
			Ser^334^	0–6
Large	**Lys^125^**	**10–17**	Glu^9^	6–9
	Glu^126^	5–9	**Thr^10^**	**9–13**
	Gly^127^	1–5	**Leu^11^**	**2–12**
	**Glu^149^**	**1–10**	Gly^14^	0–5
	**Glu^150^**	**7–14**	Asp^17^	4–6
	**Gln^153^**	**3–13**	**Tyr^49^**	**5–14**
	**Glu^154^**	**6–12**	**Pro^50^**	**12–22**
	**Val^180^**	**3–12**	**His^51^**	**6–15**
	Arg^181^	4–9	**Lys^52^**	**11–16**
	Glu^183^	0–7	**Lys^54^**	**5–13**
	**Tyr^184^**	**7–16**		

The residues assembling the majority of the interface are highlighted in bold. The columns “Seg” refers to the discrete interface segments where the listed residues are located. The column “Interface Area” displays range of interface area represented by a residue during MD simulation (see Table 6).

### Correlated Mutations of the Interface Residues

If APEX1 and pol-β evolved to form a molecular complex so that the specificity of their interaction optimized the function of the BER pathway, then one would expect that the network of inter-residue contacts constrains the protein sequence. In other words, the changes accumulated in the evolution of one of the interacting proteins would be compensated by changes in the other one [16]. Therefore, we explored whether correlated mutations in predicted interface regions between the two proteins were observed across a variety of species where the sequences of both proteins were available in the PDB.

**Figure 6:**
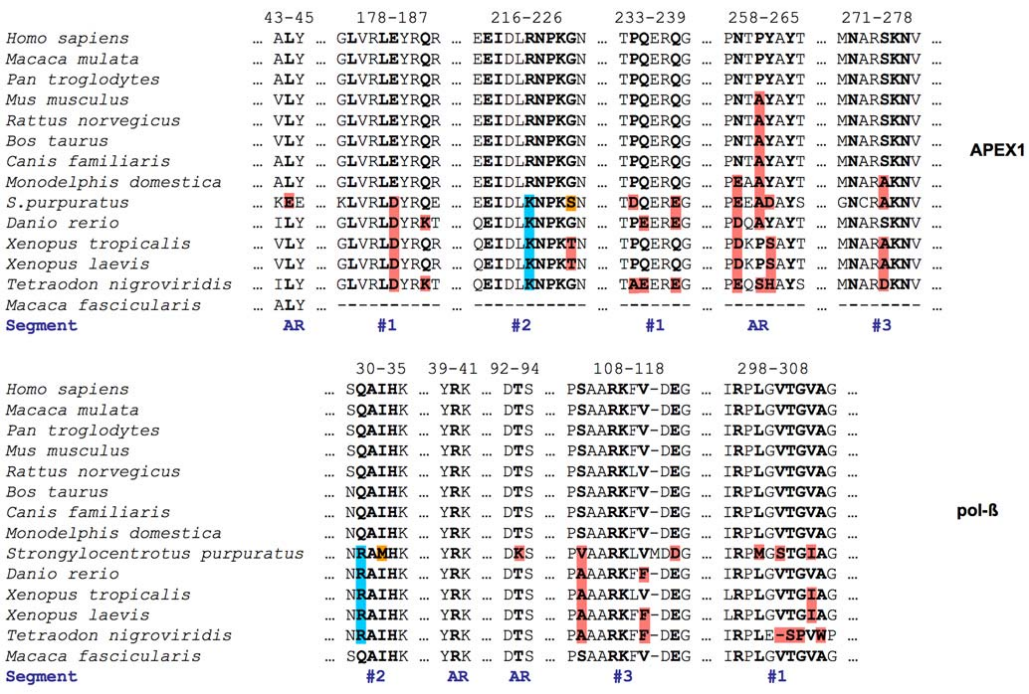
Multiple sequence alignment of APEX1 and pol-β. Only alignment for fragments of interacting regions in the 3′ complexes (with open and closed conformation of pol-β) is shown. Residues at the interfaces are in bold; neighboring residues are in normal font. Interacting residues include residues from segments #1, #2, and #3 and adjacent residues (see text), found at interface only in the complex with open conformation of pol-β. Adjacent residues are termed (where possible) AR. Correlated mutations of interacting residues are highlighted in cyan and orange. Other variations in interacting residues are highlighted in red.

In fact, multiple sequence analyses of APEX1 and pol-β revealed correlated mutation at the interface of the two proteins in the 3′-complex (Figures 3 and 6). In particular Arg^221^ of APEX1 and Gln^31^ of pol-β that interacted in the 3′-complex with pol-β in the closed conformation were changed in five organisms to Lys and Arg respectively. In four of these organisms there was also correlated variation of Ser^275^ in APEX1 and Ser^109^ in pol-β, but these residues did not interact in the predicted complex. In addition, in *S. purpuratus* there was one more coordinated change in interacting residues, Gly^225^ of APEX1 was mutated to Ser and Ile^33^ of pol-β was mutated to Met. Altogether these observations of correlated mutations provide additional support for the interactions proposed in this study.

## Discussion

In the present work we have made detailed predictions about possible interacting complexes of apurinic/apyrimidinic endonuclease (APEX1) and DNA polymerase beta (pol-β). Although it is possible that the two proteins function entirely independently of each other, our predictions were based on the assumption that at concentrations found in the nucleus the proteins interact with each other when handing off the product of APEX1 to pol-β. Experimental data indicate that for interaction to occur the two proteins have to be associated with DNA. Aligning the DNAs in the co-crystallized complexes of APEX1 and pol-β effectively positioned proteins on a DNA. Similarly, shifting the co-crystallized DNAs in either direction enabled us to orient pol-β downstream or upstream of an abasic (AP) site. Five optimal complexes were identified: two with pol-β located downstream of APEX1 (3′ to the lesion) and three with pol-β located upstream of the APEX1 (5′ to the lesion). The complexes are schematically displayed on Figure 1. Additional multiple sequence analysis of APEX1 and pol-β sequences reveals correlated mutations of predicted interacting residues in the 3′-complex, supporting the prediction. The same analysis reveals no correlated mutation in the 5′-complex.

Both 3′-complexes were energetically favorable while only one 5′-complex was stable. In particular, interacting surfaces of both proteins in the 3′-complexes open or closed conformation of pol-β repacked during MD simulation analysis to permit sufficient binding to account for complex formation. During the 1 ns of the MD simulation each complex was stable with relatively constant quantitative values of the interfaces and favorable corresponding estimated binding energies (−10 kcal/mol in each complex). On the contrary, MD for the 5′-complex with pol-β in closed conformation revealed an unstable complex that dissociated completely after 0.4 ns. Although, similar MD simulation for the 5′-complex with pol-β in open conformation revealed interactions, interface dynamics and visual inspection led us to conclude that the complex was not realistic and physical measurements were misleading. For this complex a steric trap formed in APEX and entangled a loop of pol-β (residues 299–308).

Comparison of the 3′-complexes and the weak 5′-complex with straight DNA revealed several important differences. The 3′-complexes had on average large interface areas and significantly stronger binding energies than the 5′-complex. Also the interfaces in the 3′-complexes required almost no repacking since the binding energies were low already at the beginning of the MD simulations (see Tables 1 and 3) and, therefore, the interfaces could be characterized as complementary and “ready-to-interact”. On the contrary binding energy for 5′-complex with straight DNA was very weak from the beginning and only moderately strong at the end of simulation (see Table 6).

Both APEX1 and pol-β are truncated at the N-terminus by 42 and 9 residues respectively in their crystal structures. The truncated residues would be unlikely to interfere with the predicted interacting protein surfaces in the 3′-complex. The 42 N-terminal residues of APEX1, if present, would be located at the side of the predicted interface where there is enough space to accommodate them (see Figure 2) and the missing residues in pol-β face away from the interface. In contrast, the nine missing N-terminal residues of pol-β would likely destabilize the 5′-complex as the N-terminus of pol-β is located at the interface in contact with DNA in this complex in such a tight environment (see Figure 5). This comparison provides further evidence that the 3′-complex is likely to predominate.

**Figure 7:**
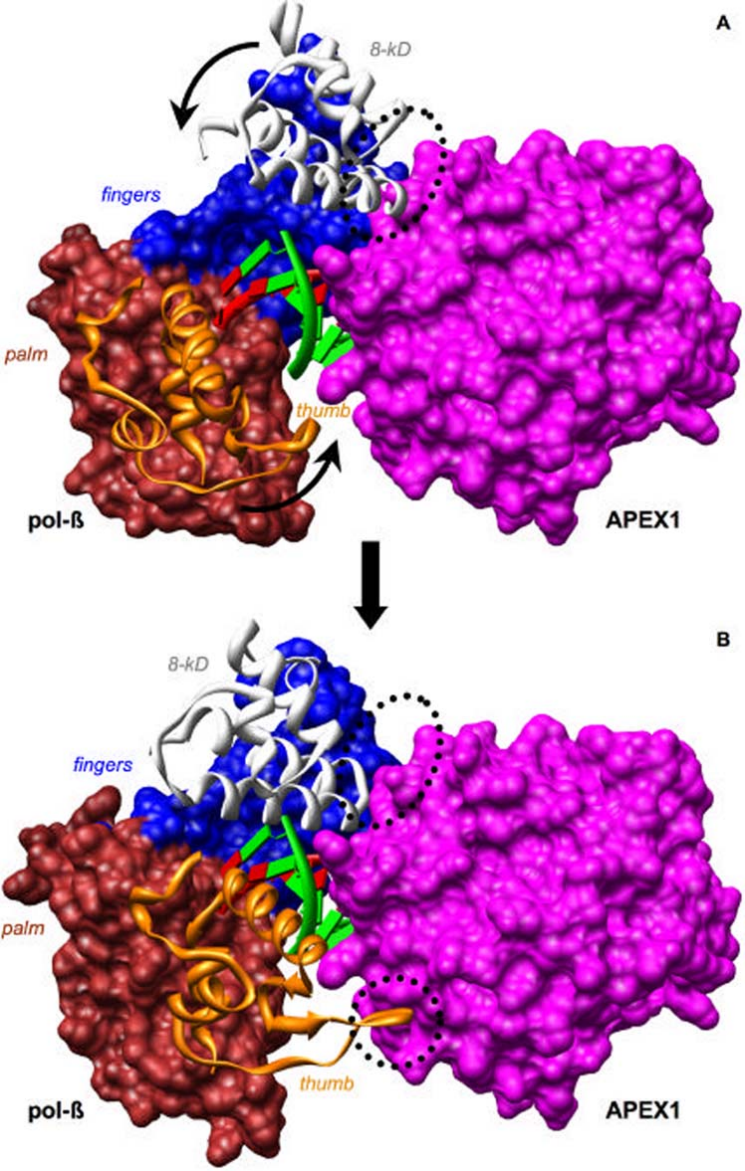
Suggested mechanism of APEX1 and pol-β interaction. Movement of thumb and 8-kDa subdomains of pol-β changes interface with APEX1 and may trigger complex dissociation. (A) shows 3′ complex APEX1 and pol-β complex with pol-β in open conformation. (B) shows the complex with pol-β in closed conformation. Area of protein-protein interface is circled. Arrows show the direction of thumb and 8-kDa subdomain movement.

Pol-β binds a cleaved AP site in open conformation [14] inserts a correct nucleotide in closed conformation and returns to the open conformation before it dissociates from the AP site. It is likely that APEX1 performs its 3′-5′ proofreading function for pol-β at this stage [17]. Pol-β then returns to the site to perform the lyase function to remove the dRP residue [18],[19]. We propose the following mechanism for APEX1 and pol-β interaction in the 3′-complex (see Figure 7). After APEX1 has cleaved the AP-site, pol-β in open conformation binds to APEX1, making a single interface comprising segments #2 and #3 and several adjacent residues including those in-between the two segments. The formed complex displaces APEX1 laterally from the cleaved site although both pol-β and APEX1 remain associated with DNA. Transition of pol-β into the closed conformation (precatalytic state) shifts the interface as movement of the 8-kDa domain splits the interface into two distinct segments #2 and #3 and weakens the interaction, while movement of the thumb introduces the new interface segment #1. Once insertion has occurred, the open conformation, still in communication with APEX1, is re-established, allowing a shift for APEX1 3′-exonuclease activity. Pol-β then returns to the site to perform its lyase function.

Although we do not see complex dissociation in our simulations, we propose two possible scenarios. In the first scenario segment #1 serves as a springboard to displace the APEX1, which eventually leads to dissociation of the complex. Since APEX1 is processive [20] it could move away from pol-β, but remain associated with DNA or alternatively it could dissociate completely from DNA. Although the complex could dissociate while pol-β is still in open conformation, such dissociation is less likely because of the larger interface area, compare to the complex when pol-β is in closed conformation. This scenario might be required when pol-β cannot perform the lyase activity and moves into long patch repair. In the second scenario, the complex does not dissociate. Instead, the proteins continue to work as a pair: APEX1 recognizes and cleaves AP sites, while pol-β inserts the incoming nucleoside and performs its lyase function. APEX1 then drags pol-β along the DNA strand to the next AP site, which pol-β is less likely to find by itself due to its transient processivity. Therefore, both scenarios imply enhanced catalytic efficiency through interaction of APEX1 and pol-β.

Of course, APEX1 and pol-β could bind and dissociate from the DNA independently from one another. However Sokhansanj et al. [21] point out that experimental data for the BER pathway indicate greater overall efficiency then can be accounted for by the individual kinetic constants of the participating enzymes. We have just provided a theoretical basis for interaction between components in one of the known subcomplexes involved in the pathway. The method described here can be used for analysis in any DNA-metabolizing pathway where weak interactions are the principal mode of cross-talk among participants and co-crystal structures of the individual proteins with DNA are available.

## Methods

### Structural Files

The structure of APEX1 was taken from PDB-file 1de8 [12], which contains APEX1 bound to abasic DNA. The structure of pol-β in the closed conformation was taken from PDB-file 2fmq [13], which contains pol-β bound to DNA in the precatalytic state. The structure of pol-β in open conformation was taken from PDB-file 9ici [22], which contains pol-β bound to DNA. The structure of template DNA has been taken from PDB-file 2ezd [23], which contains 12-mer of double stranded DNA, the longest straight piece of DNA available in the PDB.

### Superposition of DNA

Chain U of 1de8 representing the AP-site containing DNA was used to position the structure of APEX1. Chains C and D of 2fmq representing DNA with lesion were used to position the structure of pol-β. The DNA strands from crystal structures of APEX1 and pol-β identified above were aligned in order to construct possible interacting complexes. Superposition of the proteins was calculated from the alignment by minimizing RMSD between mainchain atoms of aligned nucleotides. The Kabsch algorithm [24] implemented in software Friend [25] was utilized for minimization. Chain B of 2ezd was aligned to the DNAs in pol-β co-crystal in order to extend that DNA when constructing 5′-complex with straight DNA.

### Replacement of pol-β Structure in the 5′ Complex

The length of DNA in co-crystal of pol-β in open conformation was not enough to align to the DNA in APEX1 co-crystal to construct 5′-complex. That is why the 5′-complex was constructed by replacing the structure of pol-β in the 5′-complex with pol-β in the closed conformation. Optimal position of pol-β in open conformation was calculated by aligning its structure to the structure of pol-β in the complex by using protein structure alignment method TOPOFIT [26] with RMSD of 1 Å and 198 aligned residues. Of the aligned residues, 176 were from fingers and palm subdomains, which are rigid parts of pol-β. Therefore, positioning of pol-β in open conformation was not biased by alignment of movable 8-kD and thumb subdomains.

### Molecular Dynamics Simulation and Calculation of Binding Energies

Molecular dynamics simulation was performed with the aid of the Gromacs software package [27]. The total size of the system was more than 90 thousand atoms. Atomic charges have been set by using OPLSAA force field. For abasic site atoms the charges of atom from nucleic bases have been used. The simulation was executed at 305^o^K with a time step of 1 fs. Each simulated complex included APEX1, pol-β, the DNA and explicit solvent.

### Calculation of Protein-Protein Interface Parameters

All parameters of protein-protein interfaces except that for free energy of binding were calculated using Protein-Protein interaction server [28]. The free energies of binding for protein complexes were calculated using the FOLD-X server [29] in the fashion of calculating the energies for the complex and each protein and then evaluating the free energy of binding as: ddG_(complex)_−ddG_(APEX1)_−ddG_(pol-β)_.

### Multiple Sequence Analysis of APEX1 and pol-β Proteins

BLAST [30] searches against non-redundant protein sequence database were performed by using human APEX1 (accession number NP_001632) and pol-β (accession number NP_002681) as query sequences. Fourteen eukaryotic organisms were identified where both proteins were available in each organism. The selected sequences were aligned using CLUSTALW program [31].
